# Electrophysiological effects of non-invasive Radio Electric Asymmetric Conveyor (REAC) on thalamocortical neural activities and perturbed experimental conditions

**DOI:** 10.1038/srep18200

**Published:** 2015-12-11

**Authors:** Antonio G. Zippo, Salvatore Rinaldi, Giulio Pellegata, Gian Carlo Caramenti, Maurizio Valente, Vania Fontani, Gabriele E. M. Biella

**Affiliations:** 1 Institute of Molecular Bioimaging and Physiology, Dept. of Bio-Medicine, National Research Council (CNR), LITA Bldg., Via Fratelli Cervi, 93, 20090 Segrate (Milan), Italy; 2 Department of Regenerative Medicine, Rinaldi Fontani Institute, Viale Belfiore 43, 50144 Florence, Italy; 3 Department of Neuro Psycho Physical Optimization, Rinaldi Fontani Institute, Viale Belfiore 43, 50144 Florence, Italy; 4 Research Department, Rinaldi Fontani Foundation – NPO, Viale Belfiore 43, 50144 Florence, Italy; 5 Institute of Biomedical Technology, National Research Council, (CNR), LITA Bldg., Via Fratelli Cervi, 93, 20090 Segrate (Milan), Italy

## Abstract

The microwave emitting Radio Electric Asymmetric Conveyor (REAC) is a technology able to interact with biological tissues at low emission intensity (2 mW at the emitter and 2.4 or 5.8 GHz) by inducing radiofrequency generated microcurrents. It shows remarkable biological effects at many scales from gene modulations up to functional global remodeling even in human subjects. Previous REAC experiments by functional Magnetic Resonance Imaging (fMRI) on healthy human subjects have shown deep modulations of cortical BOLD signals. In this paper we studied the effects of REAC application on spontaneous and evoked neuronal activities simultaneously recorded by microelectrode matrices from the somatosensory thalamo-cortical axis in control and chronic pain experimental animal models. We analyzed the spontaneous spiking activity and the Local Field Potentials (LFPs) before and after REAC applied with a different protocol. The single neuron spiking activities, the neuronal responses to peripheral light mechanical stimuli, the population discharge synchronies as well as the correlations and the network dynamic connectivity characteristics have been analyzed. Modulations of the neuronal frequency associated with changes of functional correlations and significant LFP temporal realignments have been diffusely observed. Analyses by topological methods have shown changes in functional connectivity with significant modifications of the network features.

The REAC technology (acronym for Radio Electric Asymmetric Conveyor) is a technology based on low intensity (2 mW at the emitter) microwave emission (2.4 or 5.8 GHz) able to interact with biological tissues by inducing radiofrequency generated microcurrents conveyed in an asymmetric manner^1^,^2^. It shows remarkable biological effects at many scales from gene modulations up to functional global remodeling of districts or in human subjects^3^,^4^,^5^,^6^,^7^,^8^,^9^,^10^. Those relevant to the central nervous system include, for instance, significant effects at the cellular stem-cell^3^,^4^,^5^ and the general levels in neurological and neurodegenerative disorders disease^6^ or in the treatment of generalized and social anxiety disorders^7^,^8^. However, we still miss a throughout biological explanation of the objective effects at tissue levels and specifically for those detected in the many neurological and psychiatric diseases where REAC appears to exert significant effects. Inferentially, these signs imply a traceable influence on brain dynamics, as those shown in previous REAC experiments on healthy human subjects showing, by functional Magnetic Resonance, (fMRI), deep modulations of motor and premotor cortex of BOLD (Blood Oxygen Level Dependent) signal diffusion in simple motor activities^9^,^10^. These effects on motor compartments prompted us to analyze the REAC potential consequences on the sensory processing exploring the outcomes of REAC application on spontaneous and evoked neuronal activities recorded at the neuronal level from the somatosensory axis of experimental male albino rats (*Rattus norvegicus* albinus). Indeed, the somatosensory thalamocortical loop of anesthetized rats represents a reasonable substrate to evaluate the influence of REAC stimulation on both the neuronal spontaneous activity and the activity evoked by peripheral tactile stimulations performed on the contralateral rat hind limb. Additionally, we performed the same experimental procedures to estimate the effects of REAC also on rats that underwent to an experimental model of chronic pain model (CP). Due to the core role of the somatosensory thalamus and cortex in CP anomalous and in normal sensory processing, the neuronal registrations have been simultaneously performed by means of two single microelectrode matrices placed, respectively, in the somatosensory thalamus and cortex. A comparison was then made between two experimental conditions, namely between data extracted from control and chronic pain animal models. To return a better characterization of the involved brain circuits, we analyzed the recorded data both from the perspective of the single neurons and from the perspective of the network of the involved neurons^11^,^12^. To this purpose we used a method previously worked out in our lab, namely an analytical framework to combine these two multiscale featuring^13^.

More specifically, we analyzed the spontaneous spiking activity and the Local Field Potentials (LFPs) before and after REAC. We applied REAC with a protocol of 100 stimuli in 5 min. Every single neuron spiking activity, the dynamic profile of the neuronal responses to peripheral light mechanical stimuli, the synchrony of discharge of neuronal small populations and the correlation index of couples of neurons, the network graph properties, such as the betweenness centrality, have been analyzed. Consistently, neuronal frequency modulations concurrent with functional correlations as well as LFP temporal realignments have been diffusely observed. Accompanying changes in connectivity as expressed by functional connectivity graphs arose with significant redistributions of the network node roles.

## Materials and Methods

### Ethical notes

All the animals have been used in accordance to the Italian and European Laws on animal treatment in Scientific Research (Italian Bioethical Committee, Law Decree on the Treatment of Animals in Research, 27 Jan 1992, No. 116). The National Research Council, where the experiments have been performed, adheres to the International Committee on Laboratory Animal Science (ICLAS) on behalf of the United Nations Educational, Scientific and Cultural Organizations (UNESCO), the Council for International Organizations of Medical Sciences (CIOMS) and the International Union of Biological Sciences (IUBS). The research has been approved by the Ministry of Health and classified as “Biella 1/2014” into the files of the Ethical Committee of the University of Milan.

### Preparation and validation of the animal models

The experiments have been carried out on male albino experimental rats (Sprague-Dawley outbred rats) 250 – 275 g at the delivery from the certified firm (Charles-River, Calco, (CO, Italy). All the animals were maintained in a 16/8 hour light-dark cycle with access to food and water ad libitum. The Chronic Constriction Injury (CCI) model (eponymously the Bennett-Xie model^14^) has been used to mimic the clinical semeiological conditions of chronic Neuropathic or Neuropathic inflammatory pain. The model is diffusely used because of its reliability and reproducibility. Briefly, after placing the animal under deep barbiturate anesthesia, (Pentobarbital [Nembutal^®^], 50 mg/kg, injected i.p), we induced a peripheral mononeuropathy by opening a surgical window over the femoral path of the sciatic nerve. After de-sheathing the muscles above the nerve, the sciatic nerve was exposed with a pair of blunted forceps delicately isolating the nerve tract and by placing four loosely constrictive ligatures around the common tract of the nerve. After suturing the surgical wound, a local antibiotic treatment was administered for three days over the margins of the wound. Repeated visual controls of the animal behavior in the first three days after the surgery have been performed to ascertain that the surgical staples had not been removed and that standard behavioral parameters were normally expressed. In comparison with control rats, the treated group showed the complete CP behavioral phenotype markers of a “painful state” within 12 to 15 days with a variable persistence of signs up to more than 1 month after surgery^15^. Specific signs and symptoms such as spontaneous postural guarding and paw withdraw during gait combined with allodynia and hyperalgesia in sensory tests were all observed and measured. Before proceeding with the recording sessions, a final behavioral validation test evaluated that all the signs of chronic pain were clearly detectable. The motor patterns were checked by a walking motor scheme test that shows the postural rhythms and patterns of paw placing during walking. Specifically, mechanical allodynia has been measured by stimulating with Von Frey filaments^16^ the plantar aspect of the treated paw (well accessible after placing the rat over a metal grid). Measures of paw withdraw latencies were then collected for all the rats every other day with double or triple tests in short time (5 minutes) intervals. Heat hyperalgesia was evaluated by the Hot plate test, again estimating by an automatic system the paw withdrawal latencies on the heated plate at 51 °C^17^. This test was repeated only at the second day after the preparation surgery and in the last day before the electrophysiological experiments, a strategy to avoid heat induced damages of the paw surface. The walking schemes have been evaluated just before the preparatory surgery and on the second and the final day before electrophysiology. Besides the walking scheme irregularities and the sensory threshold anomalies, no other evident anomalous behavioral sign was observed both in feeding or sleep rhythms.

### Experimental preparation

The rats underwent preliminary barbiturate anesthesia (Nembutal, 50 mg/kg, injected i.p) for the surgical experimental preparation of the electrophysiological experiments. The trachea was cannulated to gain direct connection to the anesthesia-ventilation device. Before the placement of electrodes, rats were paralyzed by intravenous Gallamine thriethiodide (20 mg/kg/h) injection via the dorsal caudal vein and connected to the respiratory device delivering (1 stroke/s) an Isoflurane (2.5%, 0.4 to 0.8 l/min) and Oxygen (0.15–0.2 l/min) gaseous mixture. Curarization was maintained stable throughout the whole experiment by Gallamine refracted injections (0.1 ml of the original solution/h). The anesthesia levels were maintained into ranges which prevented any corneal or retraction reflex (originally evaluated in absence of curarization), with low intensity noxious mechanical stimuli applied on a posterior paw or by scleral stimuli by fine cotton tips. An electronically regulated thermal bed maintained the rat temperature at 37.5 Celsius degrees. Refractory doses of the curarizing agent (10% of the root dose) were then delivered every 2 hours or so, to maintain at most comparable myorelaxed condition avoiding “noisy” signaling from muscle peripheral receptors.

We chose two areas for the simultaneous neuronal recordings in the left brain hemisphere (somatotopically correspondent to the right hindpaw, where the Chronic Pain model preparations were generated): the thalamic ventral-posterolateral nuclei (VPL) and the primary somatosensory (S1) cortex.

**Figure 1:**
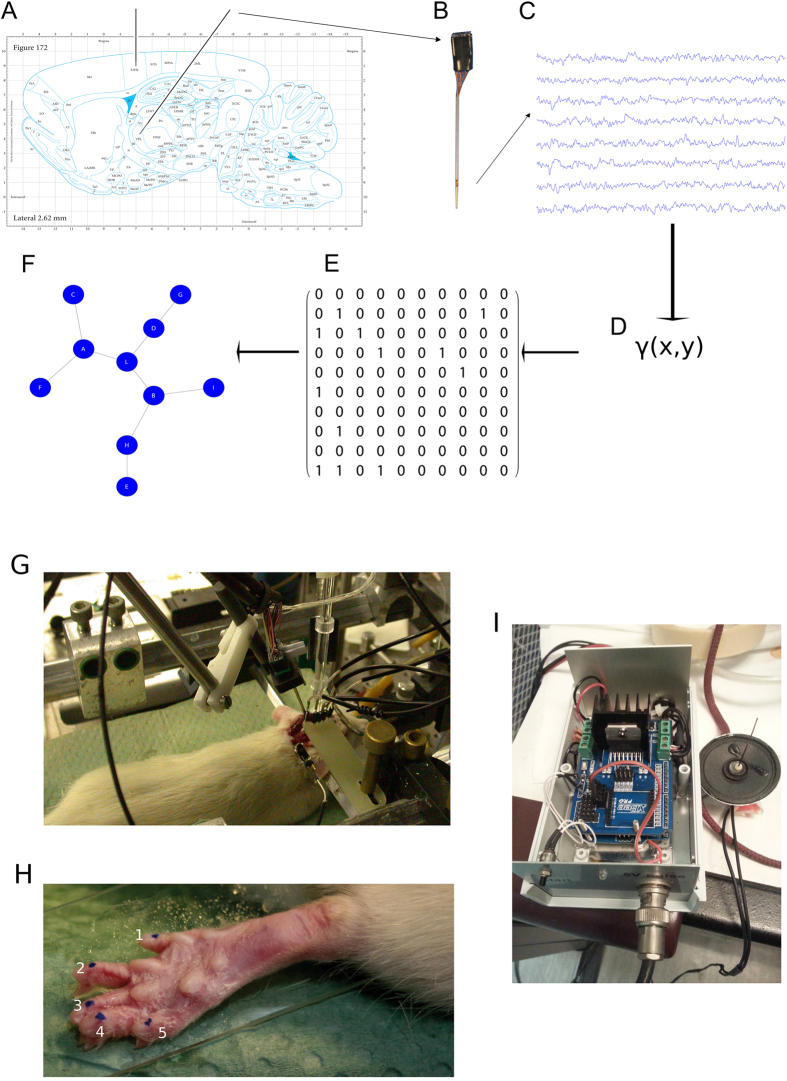
The experimental framework used in the work. (**A**) A sagittal section showing two black lines, indicating the brain regions were the multielectrode recording probes are inserted. Adapted from “The Rat Brain in Stereotaxic coordinate, 6^th^ edition”, Paxinos, G., Watson, C. (**B**) The multielectrode matrix used in the experimental recordings which produced recordings like those exemplified in (**C**). We estimated the interactions of neuronal micropopulations by applying the 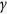 function for each electrode signal couple thus obtaining an adjacency matrix as in (**E**) that correspond to the graph in (**F**). (**G**) The anaesthetized animal with the two matrix probes inserted into the brain. (**H**) The rat right hind limb paw where the tactile stimulations were performed in the five sites (blue dots) through the device showed in (**I**).

We drilled two holes on the skull: A 3 × 2 mm bone window for the access of the cortical matrix electrodes and a larger bone window (6 × 2 mm) allowing for the simultaneous insertion of an electrode matrix directed to the thalamic Ventral-basal nuclei in slant. The neuronal recordings were obtained by using the planar electrode matrices (Fig. 1B). The planar matrices were 3 × 3 frames of tungsten or Pt-Ir electrodes, inter-tip distance 150–200 \mum, tip impedance 0.5–1 MOhm (FHC Inc., ME, USA). The cortical access was set around a reference at −1.5 mm AP and −2.5 mm ML on the left, and the electrode matrix was driven around 450 to 800 micrometers deep by an electronically controlled microstepper M238 Linear Actuator and MS208E Motor Controller (Precision Instruments, Germany.) to gain access to the cortical granular IV layer. The thalamic access was centered at the focus points of −6 mm AP, −0.8 and −2.5 mm ML. The thalamic matrix was inserted with a 25^o^ slant and driven at least to 5500 um in depth, and then slowly advanced by a second electronically driven microstepper (Precision Instruments, Germany) until clear fast responses were observed to peripheral test stimuli. All the stereotactic measures have been obtained from Paxinos-Watson atlas of the rat nervous system^18^ (see Fig. 1A,G).

### Electrophysiological recordings

Thalamic and cortical responses to fast light tactile stimuli delivered by an electromechanically regulated device on the plantar aspect of the right hind limb (see Fig. 1F) were used as anatomo-functional acceptance criterion for the electrophysiological acquisition^16^,^19^ (see below for a detailed description).

The spiking activity, along with the LFPs, has been simultaneously recorded using a wide band-pass of 1 Hz to 6 kHz allowing for the capture of both neuronal and synaptic signals.

In these recordings, as previously described, we analyzed the neuronal frequency, the responses to peripheral light mechanical stimuli, the neuronal synchronies and the thalamo-cortical correlation indices. We analyzed also some topological property of the thalamo-cortical circuit such as the betweenness centrality. The data were collected before and after the REAC application. For signal amplification and data recordings a 40 channel Cheetah Data Acquisition Hardware was used (Neuralynx, MT, USA, sampling frequency 32 kHz). More in detail, the electrophysiological signals were digitized and recorded with bandpass at 6 kHz/300 Hz for spikes, 180 Hz/1 Hz for Local Field Potentials (LFP). The data stored were analyzed off-line both using Matlab-based functions and by locally developed software^13^.

For the off-line analyses, the spikes were extracted and sorted by using the *Wave_clus* MATLAB toolbox. Sorted cells with average rates below 4 Hz and above 100 Hz were excluded from the analysis. Neurons resulted from sorting which had improbable inter-spike-interval distributions were discarded as well. LFPs were down-sampled to 0.5 KHz. After filtering and down-sampling, the spike contamination of LFP signals was null avoiding further spike removal techniques. Recorded neurons were uniformly distributed over the recording matrices and every electrode show distinct neural activity otherwise the matrix was repositioned. The timestamps of spike occurrences were represented by binary sequences where 1’s labeled a spike. We considered time bins of 1 ms thus avoiding occurrence of multiple spikes within the same bin^13^.

Finally, we split each sequence into fixed-length (from 50 to 1000 ms) overlapping windows (Fig. 1C), thus obtaining an ordered set of equal length windows.

### Functional connections by LFP phase synchrony

LFPs are low frequency signals reflecting a wide range of synaptic events. In this work, we investigated the synchrony of LFP phases originated in different recording sites during spontaneous and tactile evoked activities. We measured phase synchronies between two recorded LFP sequences (x and y) by the following function^20^


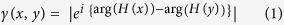


where 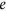 is Napier’s constant, H is Hilbert Transform, arg is the argument function and i is the imaginary unit. The Hilbert transform and the argument function were computed with, respectively, the *hilbert* and the *angle* Matlab functions. When 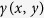 is equal to 1 (0), then 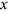 and 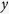 are perfectly synchronous (asynchronous).

### Complex Brain Network

By using the 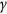 function, we estimated the functional connections of the recorded neuronal networks. We first split each recorded sequence into equal-length time windows and then we computed the adjacency matrix for all neurons or electrodes. The resulting matrices exhibited values in the unitary interval. We analyzed window sizes of 500 ms. The functional connections extracted from extracellular recordings can be represented by graphs (Fig. 1E,F).

A fixed threshold equal to a higher percentile of the weight distribution (the 75^th^ percentile) selected the strongest connections, thus allowing for the construction of the functional connection graphs^13^.

For the analysis of these graphs, we introduced a set of common statistics from the Complex Network Theory able to detect possible matches between the extracted graphs and prominent topologies. From a functional perspective, small-world networks can express two important information processing features: information integration and segregation. Functional segregation recruits specialized processing within densely interconnected nodes (cliques). Functional integration combines information processed in distributed nodes or cliques. These network properties can be measured by two statistics: the clustering coefficient (*C*) and the characteristic path length (*L*). The former measures how close the neighbors of a node are to being a clique. The latter estimates the average shortest path length in the graph, i.e. how much the nodes are accessible. Both measures, implemented in a Matlab toolbox, were used for our network analyses (clustering_coef_bu.m, charpath.m)^13^.

Ultimately, we analyzed networks that evolved in time dropping and recruiting connections and networks that come from different experimental conditions. Such a methodology requires the discussion of potential issues. First, unconnected nodes were rare but could occur after adjacency matrices were binarized. For this reason, we removed graphs in which less than the 99% of nodes were connected. Second, network statistics were applied on network with different sizes (for spiking activity) because the recording sessions returned a variable number of active neurons. However, by analyzing the observed variance of network size we concluded that C and L couldn’t be significantly affected by our network size changes. Significant changes appeared for synthetic networks that increased their size by orders of magnitude. However, we discarded graphs that were outliers (beyond 5^th^ and 95^th^ percentile) of the node, edge and density number distributions in order to obtain a better homogeneity. In the work, we refer to these two conditions as *admissibility criteria*^13^.

### Tactile Stimulation

Controlled stimuli were delivered by a blunted cactus thorn on each of five sites of the rat right hind limb (Fig. 1I,H). The tip was mounted on the dust cap of a speaker and driven by an Arduino microcontroller board (available at http://www.arduino.cc). At the beginning of each stimulation epoch the tip was lightly placed over the skin. Fast 5 ms pressure pulses were applied following a semi-random sequence. Pulses occurred in couplets. The delay between the first pulses of each couplet was set at 500 ms. Every second pulse of each couplet followed the first by a random delay extracted uniformly in the range 150–250 ms. The stimuli semi-randomness was adopted to avoid habituation phenomena of the peripheral receptors^13^,^16^.

### The REAC Application

REAC was applied either by very thin laminae of purified aluminum connected to the device by eight input channels fixed to the lamina perimeter by small crocodile-clips. Alternatively, one channel was connected to a platinum-iridium needle placed on the exposed cervical muscles (mainly on the medial raphe of the muscle acromyotrapezius). The antalgic neuro-modulation protocol was applied with 15 minutes of repeated impulses one each 1.5 s.

### Statistical Analyses

In all the analyses the non-parametric Wilcoxon ranksum test for significance has been applied. When more than two classes were considered, we applied the Bonferroni correction by multiplying the P-value for the number of classes.

## Results

We recorded the electrophysiological activity of neuronal populations from two rat brain regions, the ventroposterolateral thalamus (VPL) and the primary somatosensory cortex (S1), of two groups of animals: 12 normal rats (CR) and 15 experimental chronic pain (CCI) rats. From raw recorded signals, we extracted both the spiking activity by high-band filtering above the 300 Hz and, complementarily, the local field potentials (LFP) by keeping components below the 120 Hz. During the acute recording sessions while animals were anaesthetized, we evaluated 4 different experimental conditions, namely the spontaneous activity (rest), the tactile stimulation of rat limb paw, the REAC application in spontaneous regime and the REAC application in tactile stimulated regime. All experimental conditions were exerted on both animal groups. We performed a set of signal analyses in order to investigate the effects of REAC by considering the basilar spiking signal feature of the firing rate (FR) and spiking activity were sorted to isolate spike timings of putative neurons. In addition, we performed the analysis of the LFP synchrony that represents a measure of the coherence of the synaptic activity typically perturbed during the external-related information processing or in neurological disease^21^. The coherence between two signals is best achieved by simple difference phase metrics when acquisitions do not suffer from the problem of volume conduction as for the LFP^20^. Furthermore, by aggregating all significant LFP coherences, we operated a functional connectivity analysis of the involved thalamocortical circuit. In summary, rest activity recording sessions lasted 10 minutes and were extracted excluding the first and last minutes (becoming of 8 minutes each) while we selected the first 500 milliseconds after the onset of the stimuli as the activity evoked by the tactile impulses. Recordings with REAC protocols lasted 15 minutes and they were integrally analyzed as whole tracks.

### Spiking and LFP Activities in CP versus CCI Animals

**Figure 2:**
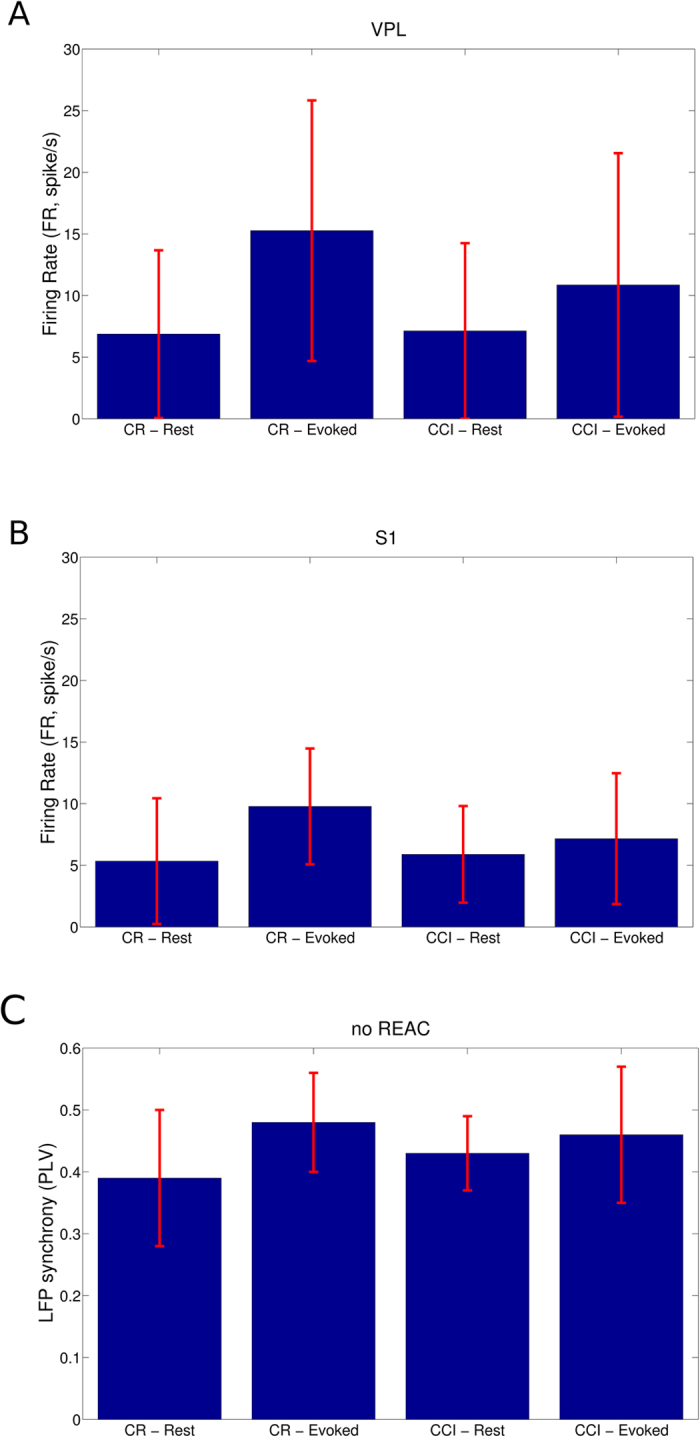
Spiking and LFP Activities in the CP and CCI groups. (**A**) The firing rate (FR), expressed in spikes per second, was estimated in the VPL thalamus in the four different experimental conditions. (**B**) The FR was estimated in the S1 cortex in the four different experimental conditions. (**C**) The average LFP phase synchrony computed in the four experimental conditions. Red lines express the standard deviations.

By analyzing the firing rate of the spontaneous activity, we found that CCI and CR animals did not differ in statistical comparisons in the activity recorded from both VPL electrodes (P = 0.533, N = 1748, ranksum test) and from S1 electrodes (P = 0.787, N = 4607, ranksum test) as shown in Fig. 2A,B.

Oppositely, by analyzing the tactile evoked firing rate activity, we found that CCI and CR animals differed both in the activity recorded from VPL electrodes (P = 0.002, N = 3058, ranksum test) and from S1 electrodes (P = 0.007, N = 8029, ranksum test) as shown in Fig. 2A,B. These results also claimed that the tactile stimulation protocol effectively elicited an increasing in the FR in both groups (CCI and CR). Indeed, hypothesis tests were significant in all 4 couples and resisted to Bonferroni correction (VPL-CR: P = 0.000, N = 2043; VPL-CCI: P = 0.001, N = 2497; S1-CR: P = 0.000, N = 4861; S1-CCI: P = 0.014, N = 5942).

In addition, by evaluating the synaptic events carried out in the LFPs we computed that the average LFP synchrony in the two animal groups and in the experimental conditions of resting and tactile stimulation. Synchrony was estimated through the phase lag value (PLV), namely a pairwise statistics that was close to 0 when signal phases are independent and 1 when signals are phase coupled. We found that within the CR group, tactile stimuli provoked a significant increment of the PLV (P = 0.000, N = 2998, ranksum test) as in the CCI group (P = 0.000, N = 2813, ranksum test). Furthermore, CCI animals showed a higher average PLV than those in CR group in the spontaneous recordings (P = 0.000, N = 2998, ranksum test) while in the tactile evoked sessions, PLV was greater in CR than in CCI animals (P = 0.037, N = 2813, ranksum test).

By assuming that LFP phase coherences represent the product of neuronal population communications^18^, we can concluded that tactile stimuli provoked an enhanced demand of neuronal communicability in order to correctly accomplish the rising information processing requirements. Similarly, the induced chronic neuropathy caused an increment of the general LFP synchrony in the somatosensory thalamocortical system in comparison to normal animals. This last fact probably shows that the neuropathy overloaded the thalamocortical system that seemingly account for less information processing in response to the incoming tactile stimulation^22^.

LFP phase coherences can be interpreted as expressions of functional connections among neuronal populations; hence, the collection of such connections constitutes a functional graph that can be analyzed though the complex network statistics. Subsequently, on these functional graphs we first computed the clustering coefficient (C, the node tendency to form highly dense clusters) and the characteristic path length (L, the average shortest path length among nodes). These represent respectively the functional counterparts of two crucial aspects of the information processing: the former indicates the tendency of the network to form clusters of nodes (functional segregation) and the latter expresses the aptitude of the network to easily transfer information among nodes.

In the LFP functional graphs, we found that C and L values were significantly different between CCI and CR animals (Fig. 2C). In particular, the C values were higher in CR than in CCI animals (P = 0.000, N = 5479, ranksum tests) and the L values were lower in CR than in CCI animals (P = 0.000, N = 5479, ranksum tests). These results indicated that thalamocortical circuits of CCI animals were worse to segregate and integrate information in comparison to CR animals. The LFP functional graphs extracted by the tactile evoked activity produced significant comparisons that highlighted the disrupting role of CP in the somatosensory thalamocortical system (see Table 1). In particular, we observed an increment of C and a decrement of L (Fig. 2C) after the onset of tactile stimuli both in the CR (C: P = 0.002, N = 2998; L: P = 0.000, N = 2998) and in CCI (C: P = 0.000, N = 2813; L: P = 0.000, N = 2998) animals. Remarkably, the extent of the C increasing was greater in CR than in CCI animals (P = 0.000, N = 5479, ranksum test) and the extent of the L decrement was smaller in CR than in CCI (P = 0.000, N = 5479, ranksum test). These topological facts showed that CP induced aberrant functional modifications in the somatosensory thalamocortical circuits that crucially affected the information processing capabilities of the involved circuits.

### REAC in Control and Chronic Pain Animals

The working hypothesis of this work was that REAC applications could show significant responses in both animal groups. In particular, we expected that REAC protocols would mainly manifest in the CCI groups were an altered physiology affected the brain regions at several perspectives (molecular, molecular, systemic, etc.). To this purpose, we repeated the same experimental sessions in conjunction with the application of the REAC protocol (see Materials and Methods section) where we applied an equivalent set of signal analyses.

**Figure 3:**
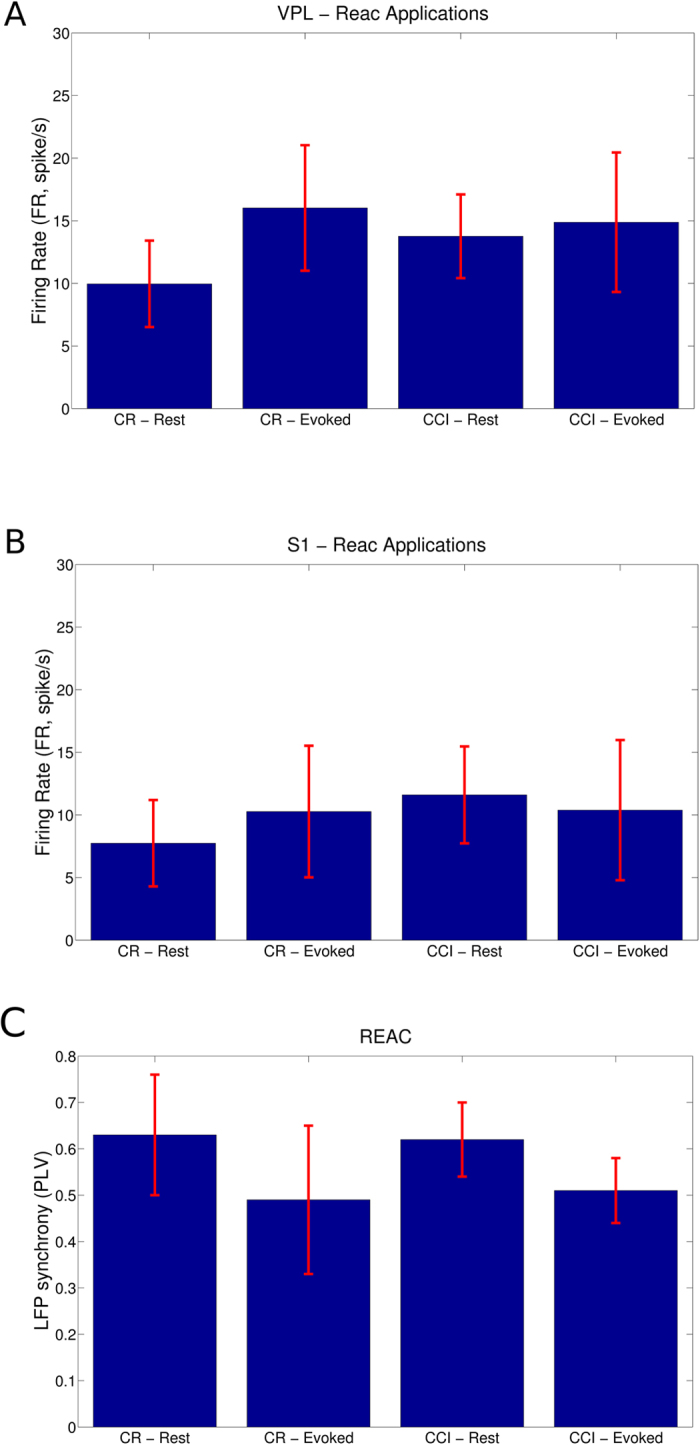
Spiking and LFP Activities in the CP and CCI groups during REAC applications. (**A**) The firing rate (FR), expressed in spikes per second, was estimated in the VPL thalamus in the four different experimental conditions. (**B**) The FR was estimated in the S1 cortex in the four different experimental conditions. (**C**) The average LFP phase synchrony computed in the four experimental conditions. Red lines express the standard deviations.

We found that REAC applications always provoked significant increments of the FR on both regions (VPL and S1, Fig. 3A,B) and in both conditions of spontaneous and tactile evoked activities (P = 0.000, N = 15632, ranksum test). Specifically, the FR in CR animals during resting state was approximately 43% higher when animals, in the same experimental conditions, underwent to REAC applications (P = 0.000, N = 3315, ranksum test) and 93% higher within the CCI group (P = 0.000, N = 4362, ranksum test). Similarly, this phenomenon was observed also during the experimental condition of combined REAC and tactile stimulation where in the CR group we found an increment of 9% (P = 0.000, N = 3315, ranksum test) and of 41% in the CCI group (P = 0.000, N = 4362, ranksum test). These results indicated that REAC applications definitely modulated the spiking activity of the neuron in the somatosensory thalamocortical circuit. These modifications appeared much more marked in the experimental chronic pain animals.

In addition, we observed that tactile stimuli effectively increased the FR in VPL both in CR group (P = 0.000, N = 2043, ranksum test) than in CCI group (P = 0.000, N = 2497, ranksum test). Remarkably this fact was also confirmed, analyzing the S1 activity, in the CR group (P = 0.008, N = 4861, ranksum test) but not in the CCI group where tactile stimulations caused a decrease of the FR (P = 0.780, N = 5942, ranksum test). These results showed that REAC interfered with the normal physiological or pathophysiological activities generally increasing the FR except when REAC protocol and tactile stimuli were combined in CCI animals.

Following the previous analysis framework, we investigated the effects of REAC applications on the LFP phase synchrony (see Table 2). We found that within the CR group, tactile stimuli provoked a significant decrement of the PLV (P = 0.000, N = 3437, ranksum test) as in the CCI group (P = 0.000, N = 4184, ranksum test). Furthermore, CCI animals showed a similar average PLV than those in CR group in the spontaneous recordings (P = 0.808, N = 3437, ranksum test) while in the tactile evoked sessions, PLV was greater in CCI than in CR animals (P = 0.136, N = 4184, ranksum test). By comparing the average LFP synchronies with those of animals with no REAC applications, we found that REAC protocol provoked a considerable increase of LFP synchronies even greater than those observed in evoked activities (P = 0.031, N = 3437 ranksum test). This fact can be considered a peculiar REAC effect expressed as a kind of information injection into the brain circuitries. This increase of injected information is even higher that that observed when a peripheral stimulus is delivered (P = 0.001, N = 3437, ranksum test)^16^,^22^

At last, we analyzed the functional connectivity of the recorded somatosensory thalamocortical circuit during REAC applications. We observed some significant modifications of the functional topological features between groups and conditions (Fig. 3C). Specifically, in resting state of CR group, functional networks became more segregated by incrementing C (P = 0.000, N = 6692, ranksum test) and more integrated by decrementing L (P = 0.000, N = 6692, ranksum test) and a similarly phenomenon was observed in the CCI group (C: P = 0.000, N = 6692, ranksum test; L: P = 0.000, N = 6692, ranksum test). Since, segregation and integration estimate the extent of the network information transfer and processing capabilities, we concluded that REAC protocols definitely augmented such efficiencies, especially in the animal affected by the neuropathy.

During the tactile stimulus sessions, functional networks appeared more integrated (P = 0.000, N = 6692, ranksum test) but equally segregated (P = 0.994, N = 6692, ranksum test) within the CR group while they were more integrated (P = 0.000, N = 6692, ranksum test) and more segregated (P = 0.000, N = 6692, ranksum test) in the CCI group. Again, we realized that REAC applications generally ameliorated the network efficiency to elaborate and transfer information, a remarkable effect mostly prominent in the CCI group where such network properties were seriously impaired by the chronic neuropathy.

## Discussion

In this paper, we report the effects of a REAC neuro-modulatory protocol application on the electrophysiological neuronal spiking and synaptic (LFP) activities recorded simultaneously from two somatosensory brain regions, namely the thalamus and primary cortex. The application of REAC on rats during the recordings showed significant and “lasting” electrophysiological effects both on normal animals as well as on an experimental model of Chronic Pain (the Chronic Constriction Injury or CCI model) yet in antiphase figure. In particular, CP animals showed significant signs of restoration of the normal thalamocortical neurophysiology after sessions of REAC applications, that is, at the best of our knowledge, the REAC application configures as the first non-invasive intervention able to modify the pathophysiology of the CP in a very short time range. Changes of CP spiking and LFP patterns appear, on the other hand, coherent with clinical findings on chronic pain^6^,^9^. More in general, REAC applications promote the synchronization of the neuronal activity, a clear sign of an augmented communication among neurons possibly able to promote the information integration necessary to boost the normal information processing of the system.

### REAC effects on the neuronal physiology

The overall results suggest profound influences on brain functional architectures at the cellular level, both in control conditions and, importantly, in strongly perturbed conditions as those accompanying sensory disorders. These findings at the neuronal scale extend the data of previous fMRI observations, significantly reporting REAC induced BOLD signal spatial reductions in motor regions activated by simple motor tasks (finger tapping or leg flexion and postural balance control^9^,^10^). These results have been interpreted as a “functional optimization of the brain structures that govern the coordination of motor control and balance”. The term “functional optimization” had, however, no current neurophysiological substrate, but being a phenomenological interpretation. The spatial BOLD signal rearrangement of motor task-activated areas may actually mirror a dynamic remodeling of the involved synaptic networks in accordance with our electrophysiological findings. In fact, BOLD signals are held as the hemodynamic counterpart of the diffuse synaptic and post-synaptic activity, sharing comparable activation/deactivation timings with the slow electrophysiological events^23^,^24^,^25^. BOLD reductions^26^ may be due either to reduction of blood flux with reduced ratio of oxygenated to de-oxygenated hemoglobin or to increase in the metabolic rate, both conditions that, increasing the paramagnetic fraction of the reduced hemoglobin also reduce the T2* signals, the source of the fMRI signal^27^,^28^,^29^,^30^,^31^. The LFP packing may appear, concurrently to BOLD spatial reductions and to microvessel distances from the synaptic spaces, energetically less expensive than expected particularly in the gamma band^32^, an advantage otherwise absent in more energy consuming regimes of natural “noisy” environments. On the other side, because LFPs represent local synaptic workloads related to BOLD activations, where spiking activities *per se* have weak or null influence^33^, it may also appear that REAC might inhibit, together with BOLD signals, the local synaptic activity. In this case, the LFP packing could display a concurrent sensible decline. As it has been shown, LFP inhibitions may appear as reduced wave powers and may be reflected in negative BOLD levels^34^,^35^. However, because BOLD mainly reflects synaptic activity, one cannot really say if the BOLD signal relates to a neural excitation or inhibition. Indeed, synaptic inhibitions are likely to anyhow reflect positive BOLD signals comparably to excitations^36^. In more recent data^37^, however, it has also been suggested that, in conditions where mainly inhibitory circuits are involved, inhibition and negative BOLD response could be related^34^. On the other side, the inhibiting central transmitter GABA doesn’t seem to be involved in significant BOLD responses confirming that the relations between inhibitory mechanisms may present complex mismatches with metabolic and flux indexes^38^. Thus, the entire picture presents severe obstacles to a monotonic interpretation. As from our experiments we cannot say if we have a LFP reduction but observe a temporal redistribution of the synaptic signals coupled to spiking rate variations in control animals, these last being not active or extremely weak in causal BOLD signal generation^23^,^39^. This would logically imply that hypothetically reduced LFP activations would have to coexist with increased spiking rates, a largely inconsistent cause-effect cascade to be prospected. In effect, a previous paper by our group found that in stimulated conditions, spike pattern prediction accuracy depended on the reliability in LFP coding for the stimulus occurrences^40^, a finding saving the strong dependence of spiking from LFPs, yet conceding that our current data are based on ongoing spontaneous activities, with potentially dissimilar LFP-Spiking coherence rules in stimulated conditions^39^. As already suggested before, the neuroelectrical composition of LFPs is extremely complex and largely unpredictable with both synaptic and extrasynaptic source^41^, such as the afferent pathways contributing to their generation, these facts knocking off true logical discernibility of LFP dynamics as dependent on specific factors or conditions. Model analyses hold that many factors may also influence the LFP recordings such as electrode geometry and recording configuration that can have a significant effect on LFP amplitude and spatial reach^42^. Indeed, the LFP heterogeneous composition of waves of different length reflect internal dynamics that are far from been clarified, though some recent papers have shown increased and decreased LFP wave components proportional to the concomitant inputs in natively multisource LFPs^43^. Alternatively, it can be held that slower intrinsic cellular events, such as K + -mediated after-hyperpolarizations can generate an extracellular field when synchronized in a population of functionally aligned cells^44^. On the other side, a hypothetical dissociation between LFPs and hemodynamic signaling appears also improbable even assuming a blood flow reduction of inhibited areas, an issue too unclear to be postulated, as observed before, even in the presence of inhibiting synapse activation workloads associated to increased BOLD. It thus seems more reasonable to think of increasingly synchronized LFPs exiting in higher spiking rates concurrently to decreased energy/blood flux requirements by the REAC induced LFP spatio-temporal reordered alignments. It may appear questionable why a “disordered” electrophysiological and hemodynamic organization could survive in contextual sensory-motor environments. It could be argued that, in the motor context, unpurposeful movements, such as those expressed in laboratory experimental conditions might tolerate larger deviations from the parsimonious dynamic schemes often seen in brain activations. However, this questionable point becomes a strong theme analyzing normal and pathological sensory neuronal behaviors.

### REAC effects on the physiopathology of chronic pain

As a rule, CCI rats show anomalous discharge patterns in a variable fraction of contralateral somatosensory thalamus and cortex neurons (namely spontaneous high discharge rate, hyperresponsiveness to light and noxious stimuli and long afterdischarges after stimulus withdrawal). These appear concurrent to the clinical signs of allodynia, hyperalgesia and persisting pain after stimulus discontinuation. In control animals, REAC generated significant increases of thalamic and cortical spontaneous spiking frequency and LFP synchronies. In CCI rats, a mean reduction of spontaneous activity frequencies in primary somatosensory thalamus and cortex were evident accompanied by LFP synchrony reduction. The chronic pain induced discharge disorders, clearly detected at the neuronal level, undergo a profound spatio-temporal reorganization after REAC just due to the LFP packing and to spiking redistributions. Specifically, these clearly recorded events could be interpreted as an abatement of noisy neuronal discharge profiles^45^,^46^. The noisy condition, widely detectable in chronic pain models, is concurrent to strong changes of functional connectivity. On the other hand, more refined mechanisms may intervene. Because LFP synchronization may be also associated to neuronal bursting that may be starters of local memories^47^, it could also be hypothesized that re-organized cortical waves might be able to instruct fast local dynamic encoding in subcortical nuclei associated to procedural memorization mechanisms. This chain of events could, again, bring to a recruitment of subcortical regions, reducing the primary area loads well imaged by a spatial reduction of BOLD signal.

## Limitations and Conclusions

Although the data framework used in this work has been successfully used in a previous work, we cannot exclude potential effects of parameter choices on results. In particular, further investigations might evaluate the role of the diverse more focused LFP frequency bandwidths since in this study we used the wide band of 1–120 Hz. For instance, REAC consequences could be more evident in narrower LFP bands, i.e. in faster synaptic events. In addition, other crucial parameters in the functional connectivity extraction should be deeper analyzed although preliminary analyses (data not shown) highlighted that the size of window (wherein the functional connections were computed) and the threshold (that binarized the functional connections) did not influence the results and the significance of statistics.

All these data tend to support the result that REAC application is able to reorganize the level of synaptic activities and the related neural circuitry dynamics in the brain. Comparable modulations of REAC emission could be detected in sensory compartments. On this track, it is convenient to add some additional note by comparing REAC induced changes on spontaneous neuronal activity with our previous results^48^ obtained from brain H_2_^15^O PETs (Positron Emission Tomography) recorded in normal subjects undergoing acupuncture applications adapt for analgesia in patients. Acupuncture provoked increased blood flux in many regions of the brain (thalamus, somatosensory cortex, cingulate cortex, cerebellum and others). In overt contradiction with the assumption of “analgesic acupuncture” the brain activated regions were overlapping those seen after thermal noxious stimuli^49^. The results were interpreted as the effect of an acupuncture induced *succedaneum* coding interfering with the idiopathic coding of noxious signals in the shared areas (namely anteromedian insula, anterior cingulate, primary and secondary somatosensory areas, cerebellum). More complex is the *a priori* that PET increased flux and increased metabolic rate, and concurrent LFP “energy expensive” synchronizations, may be assimilated, having been shown many cases of flux-metabolism dissociation. However, recent studies confirmed that acupuncture induces changes in FDG metabolism with comparable needle assets. Thus, coherence between H_2_O and FDG can be assumed, hence implying that LFP temporal reorganization might be implied as well, presuming that LFP realignments might be a first target of acupuncture too, for mere inferential logic. Increase of H2O and FDG signaling (flux and metabolism) are, however, the result of signal summation of long time lapses deeply incoherent with LFPs, developing at an order of magnitude lower and even more with spikes. However, the spiking/LFP episodes recruited by REAC are very stable and recurring in time thus assuring a coherent substrate with higher magnitude scale episodes such as blood flow and metabolic activations. More interestingly, the REAC application exerted specular effects on neuronal activities on chronic pain animals with reduction of spiking frequencies and complementary reductions of LFP dispersions echoing the quoted fMRI observations with activated area spatial reductions. This suggests, all in all, a kind of dynamic gating property of REAC that would maintain the electrogenic activity of the brain within “opportune” ranges, violated in conditions of sensory dystonias and chronic pain, and induce “recovery plans” by dynamic bidirectional rearrangements of neural dynamics.

## Additional Information

**How to cite this article**: Zippo, A. G. *et al.* Electrophysiological effects of non-invasive Radio Electric Asymmetric Conveyor (REAC) on thalamocortical neural activities and perturbed experimental conditions. *Sci. Rep.* **5**, 18200; doi: 10.1038/srep18200 (2015).

## Figures and Tables

**Table 1:** Network statistics computed on the LFP functional graphs representing an estimation of the functional connectivity of the thalamocortical circuit.

**No Reac**	**Resting**		**Tactile Evoked**	
**Network Statistics**	**C**	**L**	**C**	**L**
CR	0.416 ± 0.037	1.105 ± 0.251	0.571 ± 0.098	1.002 ± 0.194
CCI	0.312 ± 0.032	1.204 ± 0.219	0.400 ± 0.064	1.172 ± 0.201

**Table 2:** Network statistics computed on the LFP functional graphs representing an estimation of the functional connectivity of the thalamocortical circuit during REAC applications.

**REAC**	**Resting**		**Tactile Evoked**	
**Network Statistics**	**C**	**L**	**C**	**L**
CR	0.465 ± 0.057	1.104 ± 0.252	0.573 ± 0.099	0.979 ± 0.186
CCI	0.381 ± 0.049	1.204 ± 0.219	0.441 ± 0.083	1.129 ± 0.148
